# Neuro-Sweet syndrome - a rare differential diagnosis in aseptic meningoencephalitis

**DOI:** 10.1186/s42466-019-0041-1

**Published:** 2019-11-21

**Authors:** Elgin Hoffmann, Christian Boßelmann, Stephan Forchhammer, Holger Lerche, Tobias Freilinger

**Affiliations:** 1 Department of Epileptology, University Hospital for Neurology, Eberhard Karls University, Hoppe-Seyler-Str. 3, 72076 Tübingen, Germany; 2 Current address: University Hospital for Radiation Oncology, Eberhard Karls University, Hoppe-Seyler-Str. 3, 72076 Tübingen, Germany; 3 University Hospital for Dermatology, Eberhard Karls University, Liebermeisterstraße 25, 72076 Tübingen, Germany; 4 Current address: Department of Neurology, Passau Hospital, Innstraße 76, 94032 Passau, Germany

**Keywords:** Differential diagnosis to meningoencephalitis, Neuro-sweet syndrome, Neuro-Behçet disease, Paraneoplastic syndromes

## Abstract

Acute febrile neutrophilic dermatosis (Sweet‘s syndrome) is a dermatological entity, which may be associated with malignancies, drugs, and infections and which is characterized by high fever, elevated neutrophils, and tender erythematous skin lesions. Involvement of the nervous system – Neuro-Sweet syndrome (NSS) - is rare, manifesting most commonly with an encephalitic syndrome in addition to fever and dermal lesions. Here, we report an unusual case of NSS in a Caucasian male patient in the setting of B-cell-lymphocytosis, with encephalitis preceding dermal lesions. Symptoms resolved completely in response to corticoids.

NSS is a rare, but important differential diagnosis in the work-up of febrile aseptic meningoencephalitis unresponsive to anti-infectious treatment. Due to its rarity and clinical variability, diagnosis of NSS might be challenging. Knowledge of this entity may facilitate proper diagnosis and differentiation from conditions with similar clinical presentation, especially Neuro-Behçet‘s disease. It may further lead to early detection of a potentially underlying malignancy and help in initiating adequate therapy.

Dear Editors,

We would like to report a case of Neuro-Sweet syndrome (NSS) as a rare but important differential diagnosis of aseptic meningoencephalitis in the setting of B-cell-lymphocytosis, with encephalitis preceding dermal lesions.

A previously healthy 75-year-old male developed psychomotor retardation, altered state of consciousness, and fatigue for 2 weeks. There were no focal deficits, but high fever (> 39 °C) with isolated CRP-elevation (13.7 mg/dl). Erythematous plaques on both thumbs had evolved 2 days prior to presentation at the hospital (Fig. 1a). There were no oral, genital or ophthalmological lesions. cMRI was unremarkable except for leukencephalopathy. CSF analysis showed mild pleocytosis (8/μl). Microbiological/serological analysis of CSF and blood was unremarkable. Whole-body CT revealed splenomegaly and pronounced medullary cavities. Fever and CRP-elevation persisted despite calculated anti-infectious therapy, with increasing neutrophilic count (leukocytes: 8720/μl, neutrophils: 6120 /μl, 70,1% neutrophils) and dermal lesions spreading to other areas. Skin biopsy revealed neutrophilic dermal invasion without vasculitis, establishing Sweet‘s syndrome (Fig. 1b). High-dose corticoid therapy yielded complete remission of dermal and neurological manifestations. Cytogenetic investigations of bone marrow revealed B-cell lymphocytosis (continued monitoring without malignant transformation at 24 months).

**Fig. 1:**
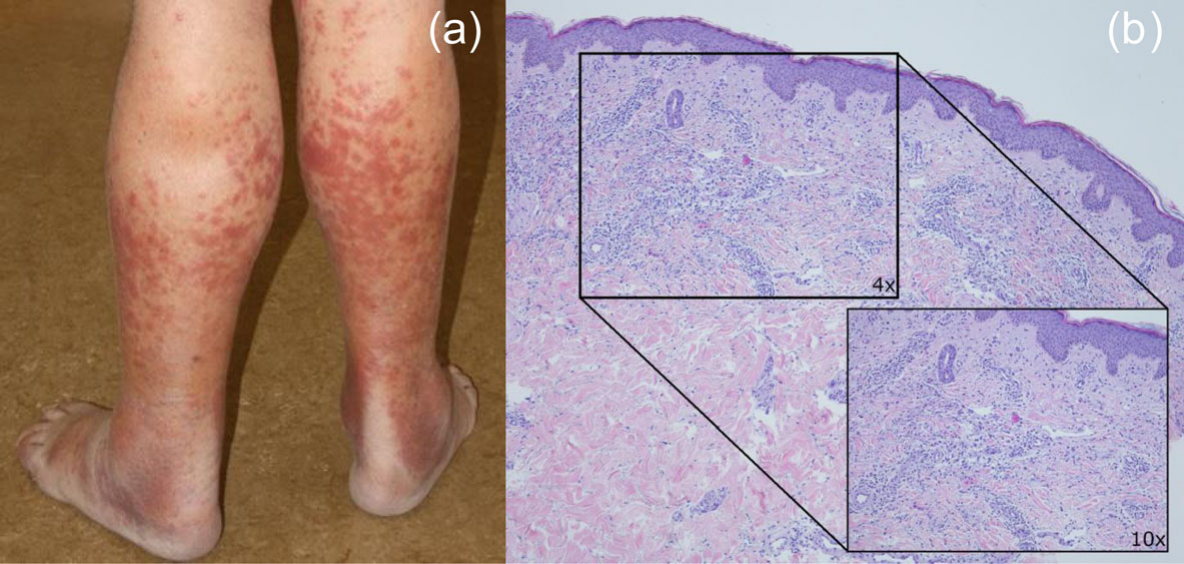
Details of dermal lesions: Close-up of the erythematous plaques (**a**). Lesions visible on the dorsal side of the calves. **b** Skin biopsy of erythematous plaques on the dorsal side of the thumbs. HE stain, 4x magnification with 10x inlay. Ribbon-like histiocytic infiltration of the upper corium with numerous neutrophilic granulocytes, granulomatous formations and edema with few perivascular lymphocytes and without vasculitis or viral cytopathic changes

Sweet‘s syndrome presents with fever, an elevated neutrophilic blood count (leukocytosis > 8000/μl or neutrophilia > 70% [8]) and tender erythematous lesions showing invasion of neutrophils with nuclear fragmentation into the dermis, typically without signs of vasculitis [3]. It can be associated with infections as well as drugs and can occur as a paraneoplastic syndrome [2]. Here, workup led to the diagnosis of B-cell leukocytosis, which can progress into chronic lymphatic leukemia. Neutrophilic invasion can affect any tissue and dermal lesions may be absent. Pathogenesis is incompletely understood, but neutrophilic invasion of the dermis and other tissues might be driven by an excessive IL-1 production in response to a stimulus (e.g. infection) [2]. As of 2017, only 69 NSS cases had been reported in the literature, preferentially affecting Japanese males in their third and fourth decade [3]. An association with HLA-B54 and Cw1 was found in Japanese patients [4, 7]. Dermal lesions can occur prior to neurological manifestation or at the same time [2, 3]. A manifestation of neurological symptoms before the onset of dermal lesions, as in our case, is rare. In addition to the features of Sweet‘s syndrome, NSS most commonly presents with an altered state of consciousness, headaches, mnestic disturbances, meningitis, and seizures but also motor deficits, dysarthria, ataxia, cranial nerve deficits, sensory impairments, and psychiatric symptoms [2, 3]. Non-enhancing hyperintense signal alterations in T2-weighted and FLAIR sequences can occur in all brain regions, especially basal ganglia, brainstem, and white matter. CSF analysis may yield an increased protein and pleocytosis with elevated lymphocytes. Clinical symptoms and imaging findings typically respond to systemic corticosteroids, although recurrence is likely [2–4].

NSS presents with a variety of neurological deficits and diagnosis may only be considered after exclusion of other neurological diseases. It shares clinical features with Neuro-Behçet disease such as dermal lesions, ulcers and neurological deficits. However, Neuro-Behçet disease shows a male predominance and younger age of onset [6]. Behçet‘s disease typically presents with uveitis, as opposed to episcleritis and conjunctivitis in Sweet‘s syndrome, and erythema nodosum-like lesions. The leukocytoclastic vasculitis and thrombosis found in Behçet‘s disease is typically absent in Sweet‘s syndrome [2]. Acute lesions in Behçet‘s disease show contrast uptake in MRI and most commonly occur in the basal ganglia and brainstem [1]. Neuro-Behçet disease also shows a different HLA association as it is consistently associated with HLA-B51 [5, 6].

NSS is an important differential diagnosis of febrile aseptic meningoencephalitis. Diagnosis is challenging due to inconsistent symptoms and imaging findings. However, knowledge of NSS and overlapping syndromes may help in initiating adequate therapy and lead to early detection of an underlying malignancy.

## Abbreviations

CRP: C-reactive protein
CSF: Cerebrospinal fluid
NSS: Neuro-Sweet syndrome
